# N-terminal tetrapeptide T/SPLH motifs contribute to multimodal activation of human TRPA1 channel

**DOI:** 10.1038/srep28700

**Published:** 2016-06-27

**Authors:** Anna Hynkova, Lenka Marsakova, Jana Vaskova, Viktorie Vlachova

**Affiliations:** 1 Department of Cellular Neurophysiology, Institute of Physiology of the Czech Academy of Sciences, Videnska 1083, 142 20 Prague 4, Czech Republic

## Abstract

Human transient receptor potential ankyrin channel 1 (TRPA1) is a polymodal sensor implicated in pain, inflammation and itching. An important locus for TRPA1 regulation is the cytoplasmic N-terminal domain, through which various exogenous electrophilic compounds such as allyl-isothiocyanate from mustard oil or cinnamaldehyde from cinnamon activate primary afferent nociceptors. This major region is comprised of a tandem set of 17 ankyrin repeats (AR1-AR17), five of them contain a strictly conserved T/SPLH tetrapeptide motif, a hallmark of an important and evolutionarily conserved contribution to conformational stability. Here, we characterize the functional consequences of putatively stabilizing and destabilizing mutations in these important structural units and identify AR2, AR6, and AR11-13 to be distinctly involved in the allosteric activation of TRPA1 by chemical irritants, cytoplasmic calcium, and membrane voltage. Considering the potential involvement of the T/SP motifs as putative phosphorylation sites, we also show that proline-directed Ser/Thr kinase CDK5 modulates the activity of TRPA1, and that T673 outside the AR-domain is its only possible target. Our data suggest that the most strictly conserved N-terminal ARs define the energetics of the TRPA1 channel gate and contribute to chemical-, calcium- and voltage-dependence.

Human transient receptor potential (TRP) subtype A1 (TRPA1) is an intrinsically cold and chemosensitive ion channel whose physiological role has been implicated in nociception, inflammatory pain, and itching^1^,^2^,^3^,^4^,^5^. This channel is one of the key sensors for various pungent and irritant compounds, being activated by thiol-reactive electrophiles and oxidants, but also by a number of nonelectrophilic chemicals, including menthol, nicotine, carvacrol, clotrimazole, and certain cannabinoids (for a review, see^6^). Moreover, TRPA1 is also dynamically modulated by permeating calcium ions^7^,^8^,^9^ and partially activated by strong depolarization^10^,^11^. While members of the TRP channel superfamily share a general tetrameric six-transmembrane (S1-S6) architecture and likely a similar mechanism of pore-opening^12^, all the stimuli that trigger this gating mechanism differ substantially and are mediated or modulated by large cytoplasmic N and C termini that flank the relatively well conserved transmembrane domain (Fig. 1). The recent high-resolution structure of TRPA1 clearly indicates that chemical signals initiated by various stimuli might indeed be readily conveyed through cytoplasmic domains to the intracellular channel gate^13^,^14^,^15^.

TRPA1 is unique among mammalian TRP channels in bearing an extensive cytoplasmic amino terminus (720 of 1119 amino acids), comprised of a prominent ankyrin repeat domain (ARD; 1–649) consisting of a tandem array of 17 ankyrin repeats (AR1–AR17), and a linker that connects ARD with the first transmembrane segment^13^,^14^,^16^. The N-terminus has been long known to be an immediate detector and integrator of the TRPA1 activation stimuli. Initial mutagenesis studies proposed that membrane-permeable electrophilic molecules such as allyl isothiocyanate (AITC) or cinnamaldehyde (CA) react with free sulfhydryl groups on specific cysteines and the primary amine of lysines (C621, C641, C665, K710 in human and C415, C422 and C622 in mouse), inducing conformational changes leading to channel opening^17^,^18^,^19^. ARD has also been thought to regulate the activity of TRPA1 from a canonical EF-hand-like Ca^2+^ binding domain beginning at aspartate D468 in AR12^7^,^8^. Although this hypothesis has been disputed by others^9^,^20^, an AR cluster centered around AR11 was shown to act as an important determinant of Ca^2+^-dependent desensitization^21^. The importance of the integrity of the ARD region was also supported by human genetic data indicating that a single nucleotide polymorphism in the *Trpa1* gene, which results in a substitution in AR4 (E179K), is associated with paradoxical heat sensation^22^,^23^.

A systematic study based on a series of chimeras between human and rattlesnake TRPA1 proposed two spatially distinct, independent and transferable AR modules, the primary module AR10–AR15, and the enhancer module AR3–AR8 (Fig. 1B), that are each capable of conferring thermal or electrophilic sensitivity to the respective, otherwise insensitive orthologue^21^. In addition, AR6 is uniquely sensitive to changes in the coupling of temperature stimuli to the channel gate, and a single-point mutation in AR6 induced temperature activation without changing the sensitivity to chemical agonists in mouse TRPA1^24^. The recently resolved high-resolution three-dimensional structure of the TRPA1 channel provides important support for the modularity of ARD by showing that AR12–AR16 are structurally integrated with the C-terminal tetrameric parallel coiled-coil, whereas the extended ARD (most likely AR1–AR11) is suspended below the membrane and forms a crescent-shaped structure^13^,^16^. All the results point to the widely accepted assumption that through evolution, the TRPA1 channel has acquired highly conserved discrete protein sub-structures that underlie its specific and species-dependent functional properties.

Recently, the N-terminus of TRPA1 has been surprisingly shown to be dispensable for a functional channel^25^. Purified human TRPA1 reconstituted into lipid bilayers can be readily activated by electrophiles, non-electrophiles as well as by cold without its N-terminal ARD (Δ1–688 TRPA1) and the channel does not seem to need Ca^2+^ or accessory proteins for its responses. Thus, the role of the N-terminus now appears to be less clear than previously assumed and may consist of forming multi-ligand binding sites, interacting with other proteins, including self-association with other non-contiguous structures of the TRPA1 channel homotetramer^26^, the proper targeting of the protein into the plasma membrane^20^,^25^, and, importantly, in the direct regulation of the channel’s gating^25^.

Structurally, the predicted ankyrin repeats (ARs) comprising the ARD N-terminal region in TRPA1 are characteristic ~33-amino acid motifs, each adopting a helix-turn-helix-extended loop secondary structure topology, stacking together in a close to linear fashion and producing a distinctive modular and elongated architecture^26^,^27^. Five of the ARs contain a strictly conserved T/SPLH tetrapeptide motif (Fig. 1A and Supplementary Fig. S1/), a hallmark of the consensus ankyrin repeat sequence contributing substantially to local conformational stability^28^,^29^. In such a motif, proline initiates the first α-helix, whereas the pair of threonine and histidine forms intra- and inter-repeat hydrogen bonds (see Fig. 2A).

In this study, we hypothesize that the strict conservation within the evolutionarily conserved T/SPLH motifs in AR2, AR6, and AR11-13 across different species suggests that an especially precisely tuned stability of these N-terminal modules is essential for the proper functioning of the TRPA1 channel. Thus, we set out to characterize the functional consequences of stabilizing and destabilizing mutations in these important structural units.

## Results

### Mutations affecting the conformational stability of ankyrin repeat 2 affect voltage-dependent TRPA1 channel gating

To examine how the conserved TPLH motif in ankyrin repeat 2 contributes to the functioning of the TRPA1 channel, we constructed three mutants: T100A, T100D, and M131G/A133T, that were intended to either destabilize or stabilize the conformation of the ankyrin repeat AR2 (Fig. 2A). We anticipated that the alanine mutation at T100 could lower the structural stability, most likely due to eliminating the intra-repeat hydrogen bonds (T100 H^N^-H103 N^δ1^, T100 O^γ1^-H103 H^N^ and T100 H^γ1^-H103 N^δ1^)^29^ and that the TPLH-mediated hydrogen-bonding network would be impacted even more by introducing a charged residue at this position. We also hypothesized that if the strictly conserved TPLH motif in AR2 contributes to channel functioning, then introducing a T/SxxH consensus sequence into the adjacent AR3 might have stabilizing effects through inter-repeat interactions, and thus be structurally and/or functionally beneficial.

To characterize the phenotypes of the mutants, we used whole-cell patch clamp recordings from transiently transfected HEK293T cells and assessed their voltage-dependent activation properties using a voltage step protocol from −80 mV to +200 mV, in 20 mV increments (Fig. 2B). The mutants expressed functional channels, however their responses to depolarizing voltage steps and their chemical sensitivities were dramatically different. Figure 2C compares the average conductance-to-voltage (*G*-*V*) relationships of voltage-dependent gating for the wild type and the mutant channels. For wild-type TRPA1, the Boltzmann fit gave a half-maximal activation voltage (*V*_50_) of 129 ± 2 mV and an apparent number of gating charges (*z*) of 0.72 ± 0.02 e_0_ (*n* = 128). The estimated *V*_50_ and *z* were neither significantly changed in T100A (135 ± 6 mV; *z* = 0.80 ± 0.09 e_0_; *n* = 9) nor in the M131G/A133T double mutant channels (124 ± 6 mV; *z* = 0.73 ± 0.04 e_0_; *n* = 7). Apparent differences, however, were seen at hyperpolarizing voltages. The T100A mutant exhibited a decreased basal conductance at negative membrane potentials, indicating a disturbed closed–open equilibrium in favor of the closed state. In contrast, the double mutation M131G/A133T resulted in a gain of function phenotype, where the channel displayed significant voltage-independent gating at negative membrane potentials (*G*_min_ of 14 ± 1% of *G*_max_; *n* = 7), suggesting that the energy required to activate the pore opening is reduced.

To test the chemical sensitivity of the mutants, we employed a standard protocol in which whole-cell membrane currents were measured first in the absence of extracellular Ca^2+^ and in the presence of the partial agonist cinnamaldehyde (CA, 100 μM for 40 s). The agonist was then washed out for 10 s, and Ca^2+^ at a concentration of 2 mM was added to the extracellular solution (Fig. 2D,E). The membrane potential was ramped up each second from –80 mV to +80 mV (1 V/s). Intracellular Ca^2+^ was buffered to low levels with 5 mM EGTA in the patch pipette to assess the effects of permeating calcium ions^9^. Such a protocol enabled us to explore the sensitivity of individual mutants not only to an electrophilic agonist, which most likely leads to the persistent activation of TRPA1^17^,^18^ but also to permeating calcium ions that activate and subsequently inactivate the channel through largely unknown mechanisms^7^,^8^,^21^. In agreement with previous reports, cinnamaldehyde at a concentration of 100 μM evoked slowly developing currents in wild-type TRPA1 (2.5 ± 0.2 nA at +80 mV after 40 s; *n* = 70) which slightly relaxed (by ~10%) to a lower sustained level upon washout. The addition of 2 mM Ca^2+^ to the bath solution induced, with a delay of ~13 s, a marked potentiation that was followed by an almost complete inactivation within 1 minute. During the 40 s-application of cinnamaldehyde, the T100A-mediated whole-cell outward currents were almost identical to those of the wild-type channels, but significantly reduced in an inward direction (Fig. 2D,F), matching the above-described reduction in voltage-induced currents at hyperpolarizing membrane potentials measured in control extracellular solution. Compared to the wild type, the delay in the onset of the second, Ca^2+^-dependent phase of the currents was almost twice as long (median value of 30 s; *n* = 9 versus 18 s for wild-type channels; *n* = 70), indicating that the reduction in inward current led to a reduction in the amount of calcium that enters the cell. The T100D mutation did not give voltage-sensitive channels in the range of voltages explored, and only four of the nine tested cells revealed detectable, but minimal, responses induced by cinnamaldehyde at positive membrane potentials (Fig. 2D). The double mutant M131G/A133T channels displayed much larger responses to cinnamaldehyde at both negative and positive holding potentials (Fig. 2E), a linear current-voltage relationship after 40 s of exposure (Fig. 2F), and about 70% voltage-independent activation (Fig. 2G). Both the delay and the degree of Ca^2+^-induced potentiation were significantly reduced compared to wild-type channels (Fig. 2E). These observations confirm the previously proposed role of the cytoplasmic N-terminal ankyrin repeat-rich region in the regulation of TRPA1 and identify AR2 to be a domain contributing to voltage-dependent gating.

### Specific mutation in conserved TPLH motif in ankyrin repeat six affects voltage- and Ca^2+^-dependent modulation

Previous findings have implicated ankyrin repeat six in determining the temperature sensitivity of mouse TRPA1 and suggested its direct role in coupling the activating stimuli to gate opening^24^. To determine the functional role of the conserved TPLH motif in AR6 of human TRPA1, we first tested the T241A and T241D mutants (Fig. 3A,B) and found them to be both functional, which suggests that strict TPLH conservation is not crucial for TRPA1 functioning. The T241D mutation caused a leftward shift in the voltage-dependent activation curve, decreasing the *V*_50_ by ~20 mV without affecting the gating charge (0.68 ± 0.06 e_0_, *n* = 16). This mutant clearly displayed significant voltage-independent gating at hyperpolarized potentials (Fig. 3B), supporting the possible role of AR6 in defining the energetics of the channel gate.

In further attempts to either strengthen or weaken the AR6 consensus sequence, we next constructed two additional mutants. The first, K239G, was anticipated to improve the ankyrin fold and allow for a more compact L shape of the repeat AR6. The second mutant, H244R, was designed to perturb AR6 by decreasing its mechanical stability^30^. Both the mutants yielded smaller currents in response to depolarizing voltages and also smaller maximal responses to chemical activators (Fig. 3C and see Fig. 4B below). This reduction, however, was probably not the result of their lower expression levels at the plasma membrane, because both the mutants displayed a significantly enhanced conductance at negative membrane potentials (*G*_min_ of 26 ± 6% and 44 ± 1% of *G*_max_; *n* = 14 and 25), a leftward shift in the half-activation potential and an increased apparent number of gating charges (Fig. 3D,E), all of which strongly support functional changes. We observed that HEK293T cells transfected with H244R exhibited constitutive inward currents after the whole-cell configuration was established at −70 mV, which indicates a constitutive activity of the mutant channel.

During the 40-s application of cinnamaldehyde, the T241D-mediated whole-cell currents were no different from those of the wild-type channels. Compared to wild-type TRPA1, the onset of the subsequent Ca^2+^-induced potentiation was not delayed, but it was apparently slowed (Fig. 4A). The average maximum rise slope of Ca^2+^-induced outward currents through the T241D channels was 0.37 ± 0.04 nA/s at +80 mV (*n* = 12), whereas in wild-type channels it was 1.2 ± 0.1 nA/s (*n* = 70). T241A was not much different from wild-type TRPA1, indicating a resilient nature of the hydrogen-bonding network in the AR6 region. In H244R, the inward currents induced after a 40-s application of cinnamaldehyde reached a similar maximum amplitude to that of the wild type (Fig. 4B,C). Both the mutations thus seemed to reduce the energy required to activate the pore opening at hyperpolarizing potentials and influence the energetic coupling of putative voltage sensor activation to gate opening. The conserved TPLH motif in AR6 has a lesser contribution to channel functioning than that in AR2 in terms of alanine or aspartate substitution. On the other hand the K239G mutation, which conforms to the consensus glycine two residues prior to the TPLH motif, had a serious mutagenic impact, most likely due to eliminating important interactions in the loop preceding AR6.

### Multimodal gating of TRPA1 depends on the stability of ankyrin repeat six

In a recently published study based on an unbiased screen of a random mutant library of mouse TRPA1, Jabba *et al*.^24^ revealed that AR6 is uniquely sensitive to changes in the coupling of temperature stimuli to the channel gate. The authors demonstrated that a single-point mutation in AR6 of mouse TRPA1 at position S250 (N249 in human TRPA1) induced temperature activation and temperature sensitivity of voltage-dependent activation, without changing the sensitivity to chemical agonists. In our study, we therefore asked whether the T241D mutant, which exhibited a depolarizing shift in voltage-dependent activation, also has a changed temperature sensitivity. We applied 3-sec heat steps from 25 °C to 50 °C (initial rate of ~25 °C s^−1^) and measured whole-cell currents from wild-type and mutant T241D channels at −80 mV and at +80 mV. As shown in Fig. 4D, both channels generated only faint currents at −80 mV. At +80 mV, increasing bath temperature elicited outward currents that persisted during heating in wild-type TRPA1, but in T241D the outward currents rapidly inactivated at temperatures above ~45 °C. In both channels, the currents exhibited only a weak temperature dependence (maximum *Q*_10_ of 2.5 in the wild type and 2.3 in T241D; Fig. 4E).

Taken together, these findings imply that destabilizing mutations in the conserved TPLH motif of AR6 create functional channels that are gated in a less voltage-dependent manner, distinctly affecting their chemical activation and Ca^2+^-dependent modulation. Our observations also support the previous identification of AR6 as a sensitive modulator of thermal activation^24^.

### Short-range stability of ankyrin repeat 12 determines the gating and calcium regulation of human TRPA1

The multiple alignment of all available TRPA1 sequences from different vertebrate species shows that the T/SPLH motifs are strictly conserved in the stretch of three adjacent repeats AR11-AR13 (Supplementary Fig. S1). In the recently resolved cryo-EM structure, this tandem repeat region is part of a crescent-shaped density assembled into a propeller-like structure that is only loosely associated with the rest of the tetrameric channel complex^13^. Such a non-canonical arrangement of the ankyrin repeat domain has been attributed to the observation that AR10 substantially deviates from the ankyrin repeat consensus and contributes to forming a sharp and flexible kink before repeat 12^16^. Indeed, in support of this prediction, we did not observe any measurable currents through the N377T/F378P channels in response to either of the stimuli when we aimed to conform to the consensus signature by mutating a highly conserved ^377^NFLH motif in AR10 (data not shown). Thus, it is conceivable that precisely tuned stability within the evolutionarily conserved T/SPLH motifs in the ankyrin repeat cluster AR11-AR13 is essential for the proper functioning of TRPA1.

To explore this hypothesis, we next assessed the consequences of individual substitutions of T415, S448 and T484 with alanine or aspartate (Fig. 5A). Only the alanine mutations were tolerated at T415 and T484, and resulted in channels with altered voltage dependency, significantly lower chemical responsiveness, and gating kinetics (Fig. 5B–E). Compared to wild-type TRPA1, these two mutants exhibited much smaller responses to cinnamaldehyde, and the addition of Ca^2+^ to the bath solution induced only a weak potentiation (Fig. 5D). Also, the full agonist allyl isothiocyanate (AITC; 100 μM) induced smaller and, in T484A, also slower responses (Fig. 5E). In agreement with previous studies, AITC elicited rapidly developing membrane currents in wild-type TRPA1, characterized by a mean time activation constant τ_on_ of 5.0 ± 0.1 s (*n* = 33). After agonist washout, the addition of 2 mM Ca^2+^ to the bath solution induced a marked inactivation (to 53 ± 5% of maximal response, after 100 s). The average AITC-induced currents through T415A were smaller, but τ_on_ and the extent of Ca^2+^-dependent inactivation were not significantly different from wild-type channels (4.7 ± 0.2 s; to 42 ± 6% of maximal response; *n* = 16). In contrast, T484A-mediated currents exhibited a mean time activation constant τ_on_ of 12.6 ± 0.4 s (*n* = 10) and almost complete Ca^2+^-dependent inactivation (19 ± 4%). The lower sensitivity of T484A is also visible in the higher rectification ratio during a 40-s application of AITC (Fig. 5E, lower graph). This result indicates that T484 in AR13 is structurally required for the normal functioning of the TRPA1 channel.

In S448A and S448D, AITC failed to induce any appreciable currents at any examined membrane potential (Fig. 6B), indicating that this strictly conserved serine is crucial either for conformational stability, function or expression. To distinguish between these possibilities, we aimed to improve the stability of AR12 by constructing two consensus-based mutations, S448T and K446G. We hypothesized that if S448 contributes substantially to ARD stability through intra- and inter-repeat hydrogen bonding, then the threonine at this position could improve ancillary interactions in its microenvironment by hydrophobic interactions associated with the threonine methyl group^29^. The second mutation introducing glycine at position K446 was anticipated to better stabilize the ankyrin fold by stabilizing its L-shaped conformation and further allowing for a better intrinsic stability of AR12 and the interfacial stability between neighboring ARs. Notably, both the mutations produced functional channels that exhibited greater currents in response to depolarizing voltage steps and a significant conductance at negative membrane potentials, indicating the presence of a strong voltage-independent component (Fig. 5F,G). Moreover, we noticed that HEK293T cells transfected with K446G (but not with S448T) exhibited a much greater extent of cell rounding and detachment than cells expressing wild-type channels, which indicates a constitutive activity of the mutant channel (Supplementary Fig. S2). The estimated *V*_50_ and *z* were not significantly different from wild-type TRPA1, being 122 ± 4 mV and 122 ± 8 mV and 0.66 ± 0.04 e_0_ and 0.64 ± 0.04 e_0_ for K446G and S448T (*n* = 14 and 19). A bar graph in Fig. 5G compares the average values of the voltage-independent component (*G*_min_/*G*_max_) for the examined T/SPLH mutants and suggests that the stretch of canonical ankyrin repeats AR11-13 tightly modulates the transition between the closed and open state of the channel in a manner that is independent of voltage sensor activation.

A remarkable finding was an apparent difference between the characteristic currents induced by cinnamaldehyde and AITC in S448T and K446G (Fig. 6A,B). The currents induced by 100 μM cinnamaldehyde were significantly greater and, upon the addition of Ca^2+^ to the bath solution, reached amplitudes that were ~1.4-fold and 1.7-fold the amplitude of the wild-type channels. The currents resembled wild-type TRPA1 in terms of the extent of Ca^2+^-dependent desensitization (Fig. 6A). In a striking contrast, the maximum currents induced by AITC were smaller than in the wild-type channels and exhibited only a slight calcium-dependent desensitization (to 72 ± 5% and 84 ± 6% of the maximal response; *n* = 6 and 10; Fig. 6B).

Although the voltage causing half-maximal activation (*V*_50_) in S448T was not different from the wild type, the voltage-independent component of cinnamaldehyde-induced activation was different (Fig. 6C). Figure 6D shows the average conductance-to-voltage (*G*-*V*) relationships of voltage-dependent gating calculated by normalizing *G*-*V* curves with the *G*_max_ value obtained after a 40-s exposure to 100 μM cinnamaldehyde. The S448T mutant clearly exhibits a much stronger voltage-independent component of gating at negative membrane potentials, indicating that cinnamaldehyde alone, which is a sub-maximal stimulus for TRPA1, is sufficient to produce almost voltage-independent gating in S448T.

Taken together, our data support the previous suggestion that calcium-dependent desensitization of AITC-induced responses is specified by an AR cluster centered around AR11^21^. Moreover, we show that subtle changes in AR12 stability affect the Ca^2+^-dependent desensitization to varying degrees according to the mode of chemical activation, and increase the voltage-independent component of TRPA1 channel gating.

### Strict conservation of the T/SPLH motifs in AR11-AR13 are required for functional interactions and not for targeting TRPA1 to the plasma membrane

Our results demonstrate that the strictly conserved T/SPLH motifs are distinctly involved in multimodal activation of human TRPA1 channel, and mutations at these positions may perturb intra- or inter-domain interactions, leading to altered activation profiles. We show that a subtle change in the structural stability of the AR11-13 stretch leads to qualitative changes in voltage-, cinnamaldehyde- and AITC-induced gating. On the other hand, changes in plasma membrane expression may also affect the qualitative behavior of the TRPA1 channel^23^,^31^. The increase in current responses in the S448T construct was surprising, since threonine is a conservative substitution, therefore we assessed the extent to which the mutation’s effects might reflect changes in surface expression. We used cell-surface biotinylation assay to examine the surface expression of the wild-type and two mutant proteins: the gain-of-function mutant S448T, and T415A as a loss-of-function control. As shown in Supplementary Figure S2, immunoblots of S448T and T415A confirmed that the proteins were processed and targeted to the membrane to a comparable extent to that of wild-type TRPA1. Thus, the changes in functionality are most likely due to structural changes. Additional support for this hypothesis stems from the bi-directional scatter plots shown in Fig. 6E,F. These graphs correlate, for each mutant, the maximum conductances induced by depolarizing voltage (+200 mV) against the maximum responses induced by cinnamaldehyde or AITC. Voltage, cinnamaldehyde and intracellular calcium, which are all sub-maximal stimuli for TRPA1, interact allosterically to enhance channel opening. The observation that alanine substitution at threonine T100 diverges from a linear relationship suggests the involvement of this residue in coupling the voltage-sensing domain to TRPA1 channel opening. On the other hand, AITC is a full agonist of TRPA1, and mutations within the ankyrin repeat stretch AR11-AR13 seem to primarily affect the energetics of gating in the absence of membrane depolarization (seen in Fig. 5F,G), and coupling between the AITC-interacting region and the channel gate (seen in Figs 5E and 6B).

### Conserved T/SPLH motifs as putative phosphorylation sites

In general, the variable molecular surfaces formed by the assembly of ankyrin repeats and their modular architecture render AR proteins highly versatile and prone to binding with other biologically important proteins^26^,^32^. The extensive N-terminal ankyrin repeat domain of TRPA1 has been proposed to interact with several proteins that modulate the channel’s functioning, cytoplasmic levels, turnover, or trafficking^31^,^33^,^34^,^35^. Evidence has also been presented that the N-terminus may be involved in phosphorylation-mediated regulation of the channel downstream of the phospholipase C and Src tyrosine kinase signaling pathways^36^,^37^. In the context of our study, we considered that some of the serines or threonines from the N-terminal T/SPLH motifs may represent potential phosphorylation sites, particularly for proline-directed Ser/Thr kinases. Among these, cyclin-dependent kinase 5 (CDK5) is a neuron-specific kinase of great functional relevance, known to regulate nociceptive signaling via the N-terminus of the TRPA1-related channel TRPV1^38^,^39^. Bioinformatics analysis of the human TRPA1 primary sequence predicts T100, T241, T415, S448, and T484 to be consensus sites for CDK5 at a high stringency level, some of these sites with even better scores than that predicted for threonine T407 in human TRPV1, the residue which is directly phosphorylated by CDK5^38^ (Supplementary Table S1).

At first sight, the serine and threonine residues constituting the conserved T/SPLH motifs examined above are not likely to be involved in the phosphorylation of TRPA1, because the phosphonull alanine mutations and phosphorylation mimicking aspartate mutations did not lead to opposite changes in the functioning of the channel. However, structural changes around these residues, such as the M131G/A133T mutation, may affect phosphorylation at the contiguous sites - in a constitutive or activity-dependent manner. At the same time, the prediction shows that threonine substitution at S448 may also affect the prediction score. Therefore, as the first logical step, we tested the relevance of these considerations by assessing whether the co-expression of TRPA1 with CDK5 and p35, a CDK5-specific activator, may modulate voltage-dependent channel activation. As shown in Fig. 7A,B, co-expression of CDK5/p35 resulted in no change in the maximal responses to depolarizing voltage, and only a slight increase in voltage-independent gating at hyperpolarized potentials. Similar effects have been also observed in cells co-expressing TRPA1 with p35 alone, but not in those expressing TRPA1 with CDK5. The co-expression of p35 alone or with CDK5 significantly increased TRPA1-mediated responses to cinnamaldehyde (Fig. 7C,D). Some authors suggest that CDK5 is present and functionally relevant in HEK293 cells^40^, while others have reported that HEK293 cells only express CDK5 at low levels and they lack endogenous CDK5 activity^41^. Thus, our result indicates that TRPA1 may be a substrate for the CDK5/p35 complex and/or its interaction with p35 may stabilize the activated state.

Proteolytic cleavage of p35 to p25 results in the cytosolic redistribution of CDK5, and therefore we also tested the effects of CDK5/p25 co-expression on TRPA1. Generally, we observed clear hallmarks of cytotoxicity in cells overexpressing p25, with or without TRPA1, which is an observation consistent with the literature^42^. The co-expression of TRPA1 with CDK5/p25 induced a less significant toxicity than that seen when TRPA1 was co-expressed with p25 alone, an explanation for this could be that the inclusion of CDK5 partially buffered the detrimental effects mediated by p25 alone. Intriguingly, CDK5 alone led to an increased responsiveness of TRPA1 to cinnamaldehyde, but co-transfection with p25 abolished this effect (Fig. 7E,F). Thus, given these findings, we cannot unambiguously exclude some specific effects of CDK5.

Within the N-terminal region of TRPA1, a sequence prediction analysis also reveals three additional putative phosphorylation sites for CDK5, containing S344, S616 and T673 (as shown in Table S1). To test the functional role of these residues and to obtain a complete picture of the role of the T/SP motifs in the N-terminus of TRPA1, we constructed six additional mutants in which either serine or threonine were replaced by either alanine or aspartate to mimic the non-phosphorylated and phosphorylated forms of the TRPA1 protein, respectively. As shown in Fig. 8, only two mutations caused significant changes in TRPA1 channel’s activity: the S344D mutation failed to produce any appreciable currents, suggesting either a structural disturbance around the AR9 repeat or a failure of functional expression as a specific result of the phosphomimicking substitution. On the other hand, the T673D mutation resulted in channels whose responses to cinnamaldehyde were increased almost threefold (from 2.4 ± 0.2 nA to 6.6 ± 1 nA at +80 mV; *n* = 70 and *n* = 8). According to the recently resolved TRPA1 structure, T673 is solvent-accessible and located in a flexible loop connecting the β-strands to the helix-turn-helix motif preceding the pre-S1 helix, i.e., well situated in a locus especially important for the detection, integration and transmission of activation stimuli^13^. This, together with our finding that the mutant T673A channels remained largely unchanged, indicates that this location, the resolution of which would require more detailed measurements and modeling, may indeed represent a candidate target for Ser/Thr phosphorylation and further studies are needed to explore this possibility.

## Discussion

A central observation of this study is that the most conserved N-terminal consensus T/SPLH tetrapeptide motifs, which initiate the helix–turn–helix conformation of the repeats AR2, AR6, AR11, AR12 and AR13, are required for the proper functioning of TRPA1 and distinctly contribute to its multimodal activation. The extensive N-terminal ankyrin repeat domain, according to which this channel has been dubbed, represents almost 74% of the cytoplasmic portion and 57% of the whole protein. Although the N-terminus is likely to be dispensable for a functional channel^25^, it is hard to believe that evolution has allocated such a large and structurally organized proportion of a protein merely for auxiliary functions such as trafficking, subcellular localization, homotetramerization, or a number of poly-specific interactions. Indeed, for examples, AR6 dictates the directionality of temperature activation to mouse TRPA1^24^ and two isoforms of a *Drosophila melanogaster* TRPA1 ortholog, which arise from alternative splicing of the N-terminal region, enable flies to detect electrophilic compounds, but only one of them is highly sensitive to temperature^43^.

It has been well demonstrated that the two N-terminal AR modules, the primary AR10-AR15 and the enhancer AR3-AR8, are independent and transferable, each capable of bestowing thermal or electrophilic sensitivity to the human TRPA1 channel^21^. However the extent to which the individual ankyrin repeats correspond to functional units still needs to be fully elucidated and understood. Previous mutagenesis and chimaeric studies have identified regions within the N-terminal ARD that dictate the pronounced specificity of the temperature, electrophilic, non-electrophilic, and oxidative sensitivity of TRPA1^7^,^8^,^17^,^18^,^24^,^44^. Mutations within the ARD frequently rendered the TRPA1 channel non-functional and any mutation of AR consensus residues had been thought to result in an unstable protein^26^. To theoretically estimate the putative effects of mutations on the stability of ARD, we used the program FoldX that calculates the folding energies of proteins and the effects of a point mutation on the stability of a protein (see Supplementary Methods). Theoretical energy measurements around ankyrin repeat 2 predicted the average free energy change ΔΔG = 1.05 kcal/mol for T100A and 4.63 kcal/mol for T100D, indicating the respective smaller and greater destabilizing effects of the mutations. In contrast, the double mutant M131G/A133T resulted in the average free energy change ΔΔG = −5.61 kcal/mol. These predictions correlate very well with the functional profiles shown in Fig. 2.

The putatively destabilizing mutations in AR6, T241A and T241D, had pronounced effects on the estimated free energy changes (ΔΔG = 1.74 and 5.53 kcal/mol). However, both of these mutants were functional. On the other hand, mutations predicted to either destabilize or stabilize AR6 (H244R; ΔΔG = 2.95 kcal/mol and K239G; ΔΔG = −3.61 kcal/mol) led to similar phenotypes with constitutive activity and changes in voltage dependence (Fig. 3C–E), suggesting that AR6 contributes to the energetics of the channel gate under resting conditions. The functional role of the extended loop preceding AR6 appears to be more important than the TPLH-mediated hydrogen-bonding network. When mutated, this region may sterically hinder and destabilize the channel gate upon depolarization. The T241D mutation, expected to be the most disruptive, changed the parameters of Ca^2+^-dependent potentiation (Fig. 4A) which may reflect perturbations in coupling between the calcium sensing domain and gate opening.

**Figure 1:**
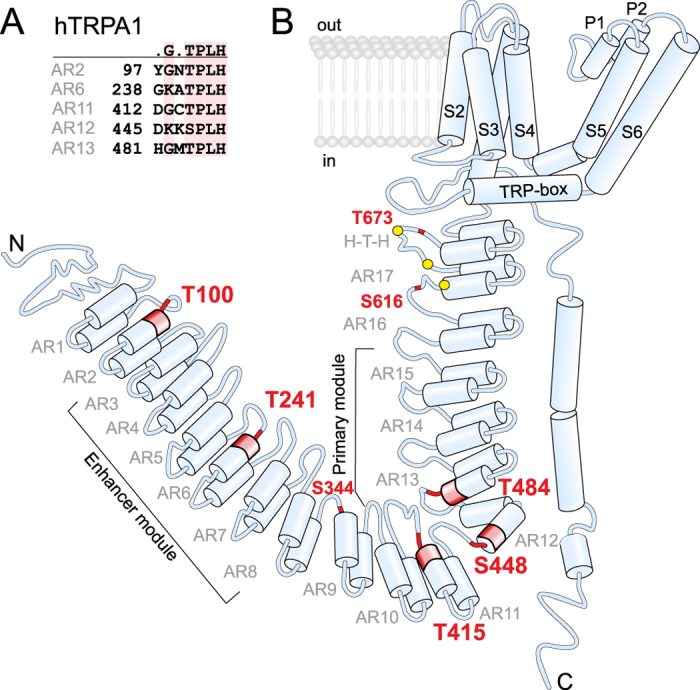
Topology of human TRPA1 channel subunit. (**A**) Sequence alignment of highly conserved T/SPLH motifs in the N-terminal ankyrin repeats (AR) of human TRPA1 (hTRPA1). (**B**) Schematic of one hTRPA1 channel subunit (according to Paulsen *et al*.^13^,). TRP-box denotes TRP-like domain, H-T-H indicates helix-turn-helix motif. Conserved threonines and serine from T/SPLH tetrapeptide motifs and T/S residues mutated in this study are indicated in red, reactive cysteine residues (C621, C641 and C665) are represented by yellow circles.

**Figure 2:**
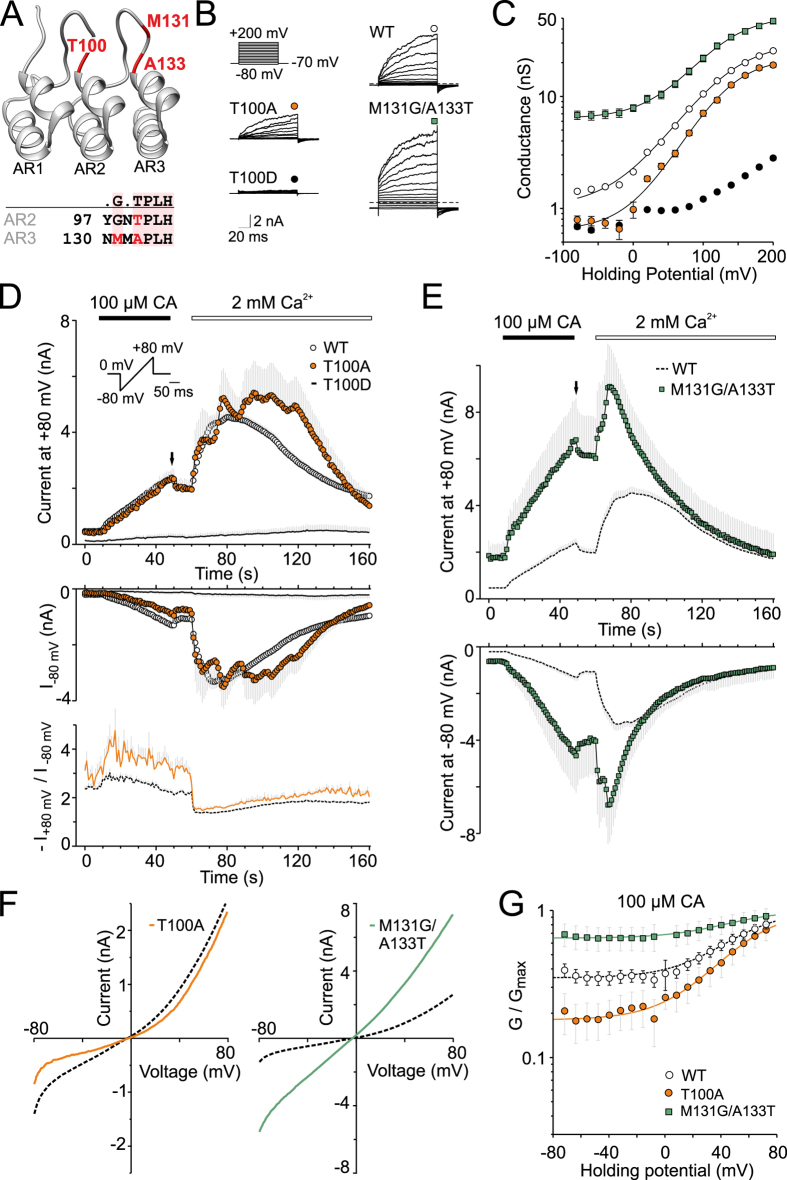
Mutations affecting the conformational stability of the ankyrin repeat 2 and 3 affect voltage-dependent TRPA1 channel gating. (**A**) Homology model of the first three N-terminal ankyrin repeats (AR) of hTRPA1 based on human ankyrinR (pdb code 1N11). In the consensus T/SPLH motif, the hydroxyl from the threonine/serine side-chain forms a hydrogen bond with a nitrogen atom from the histidine side-chain and results in a sharp turn prior to the first helix in the AR. Glycine two residues prior to T/SPLH allows for the compact L shape of the AR. Residues mutated in this study are indicated in red. (**B**) Representative current traces in response to indicated voltage step protocol (100 ms voltage steps from −80 mV to +200 mV; increment +20 mV; holding potential −70 mV) recorded in control extracellular solution ~1 min after whole-cell formation. (**C**) Average conductances obtained from voltage-step protocols as in B. Data represent the means ± S.E.M. (*n* = 128 for WT and 7–26 for mutants), solid lines are best fits to a Boltzmann function as described in Methods. In some cases, the error bars are smaller than the symbol. (**D**) Time course of average currents through wild-type (solid circles, *n* = 70), T100A (filled circles, *n* = 9) and T100D (solid line, *n* = 9) mutants measured at +80 mV and −80 mV. The average currents are shown with gray bars indicating S.E.M. The application of 100 μM cinnamaldehyde (CA) and subsequent addition of 2 mM Ca^2+^ are indicated above. Below, average rectification of currents shown above, plotted as a function of time. Inset: voltage-ramp protocol. (**E**) Time course of average CA-induced currents in M131G/A133T. The average current for WT is shown in dashed line with gray bars indicating S.E.M. (**F**) Average current-voltage relationships of traces measured at times indicated by an arrow in panels D and E for wild-type (dashed line) and indicated mutants. (**G**) Voltage-independent gating in M131G/A133T. Average conductances obtained from voltage ramp protocol (shown in panel D) normalized to *G*_max_. Dashed line for wild-type and solid lines for mutants are best fits to a Boltzmann function.

**Figure 3:**
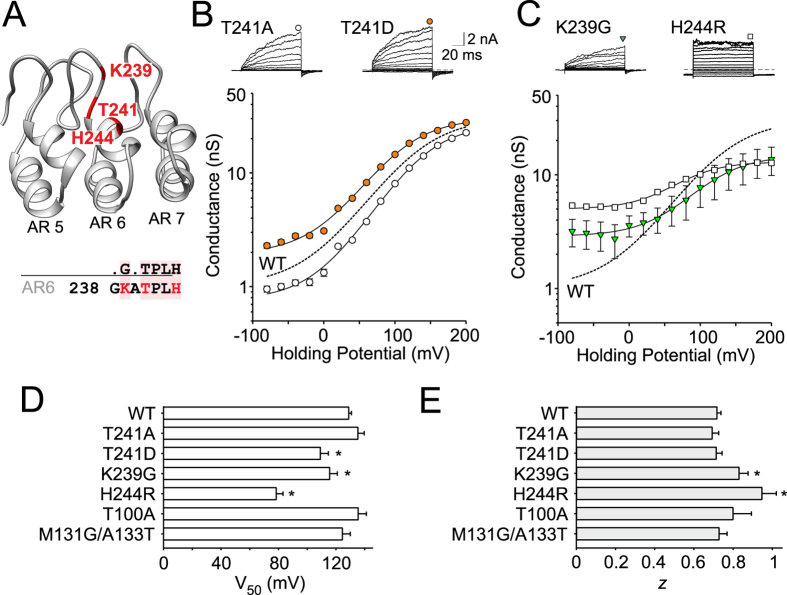
Specific mutation in the conserved TPLH motif in the ankyrin repeat six affects voltage-dependent modulation. (**A**) Homology model of the ankyrin repeats 5 to 7 of hTRPA1 based on human ankyrinR (pdb code 1N11). Residues mutated in this study are indicated in red. (**B**,**C**) Representative current traces in response to voltage step protocol (holding potential −70 mV; 100 ms voltage steps from −80 mV to +200 mV; increment +20 mV) recorded in control extracellular solution ~1 min after whole-cell formation. Representative current traces for wild-type TRPA1 are shown in Fig. 2B. The H244R mutation produced non-zero current at −70 mV at the beginning and the end of each voltage step during the voltage-step protocol (the dashed line depicts the −70 mV baseline), indicating the constitutive activity. Below, average conductances obtained from voltage step protocols shown above. Solid lines are best fits to a Boltzmann function. The dashed line represents the fit obtained from data for wild-type TRPA1 shown in Fig. 2C. Note, both mutations, the stabilizing K239G and destabilizing H244R, reduce the responses of TRPA1 to voltage. (**D**) Summary of half-maximal activation voltage (*V*_*50*_) of wild-type hTRPA1 and individual mutants from experiments as in (**B**,**C**) and Fig. 2C. The asterisks indicate significant differences between mutant and wild-type TRPA1 (*P < 0.05, *n* = 138 for WT and 7–27 for mutant channels). (**E**) Summary of apparent number of gating charges (*z*) of wild-type hTRPA1 and individual mutants from experiments as in (**B**,**C**) and Fig. 2C. The asterisks indicate significant difference from wild-type TRPA1 (*P < 0.05).

**Figure 4:**
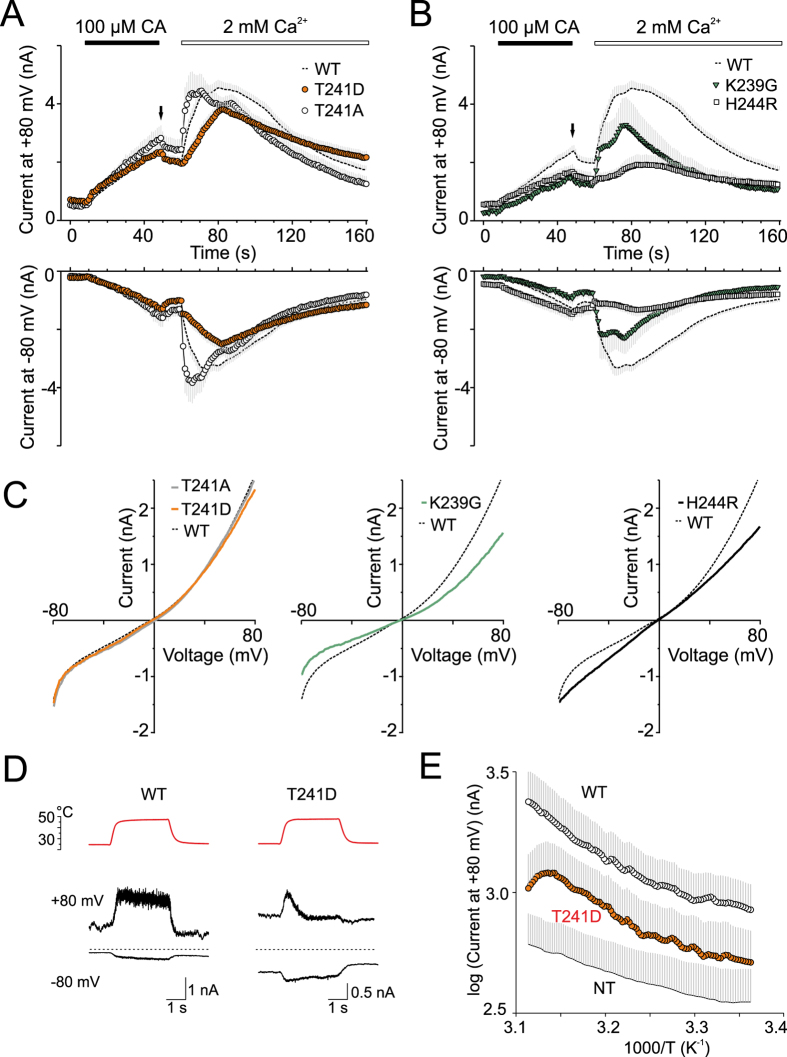
Multimodal gating of TRPA1 depends on the stability of ankyrin repeat 6. (**A**,**B**) Time course of average whole-cell currents induced by 100 μM cinnamaldehyde (CA) in Ca^2+^-free solution and then exposed to 2 mM Ca^2+^, measured at +80 mV and at −80 mV in wild-type (dashed line) and indicated mutants. The application of CA and subsequent addition of 2 mM Ca^2+^ are indicated above. (**C**) Average current-voltage relationships of traces measured at times indicated by an arrow in panels A and B for wild-type (dashed line) and individual mutants T241A (gray line), T241D (orange line), K239G (green line) and H244R (black solid line). (**D**) Time course of representative whole-cell current responses to application of 25–47 °C heat step recorded from cells expressing wild-type and mutant hTRPA1 at +80 mV and −80 mV as indicated. The upper row of records (red lines) shows the temperatures of superfusing solution (ECS) measured by a thermocouple inserted into the shared outlet capillary of the drug application system. (**E**) Arrhenius plot of average heat-induced whole-cell currents obtained from wild-type TRPA1 (*n* = 9), the T241D mutant (*n* = 7) and from non-transfected HEK293T cells (NT, *n* = 6) measured at +80 mV.

The recent study by Moparthi *et al*.^25^ implicated the N-terminal ARD region as an important gating modifier that may regulate the channel’s behavior in a voltage-dependent manner. The ankyrin repeats around AR2 and AR6 are sequentially distant from the transmembrane region expected to ‘sense’ the membrane electrical field, but they may contribute to voltage-dependent gating through interactions with the TRPA1 protein itself, membrane or membrane-associated factors^13^,^16^. The recently resolved structure of TRPA1 provides a mechanistic explanation of how the proximal part of ARD can communicate with the channel gate^13^: the information from the ARD can be transduced through the overlying helix-turn-helix motif of the linker region that forms a network of packed interactions with the TRP-like domain (Fig. 1B). From the structure, it is also apparent that the mutational consequences for the canonical ankyrin cluster AR11-AR13 cannot be easily interpreted, because the region including AR11 is represented by only a weak electron density map and contains a flexible kink before AR12. If the T/SPLH motifs in AR11-AR13 assumed an ideal conformation in the context of the tertiary fold, the alanine and aspartate mutations at T415 would consistently change the free energy by −0.6 and 3.2 kcal/mol and would lead to only moderate changes at T484 (ΔΔG from −1.03 to 1.36 kcal/mol). The conservative substitution S448T would yield ΔΔG = −1.12 kcal/mol, i.e. it should have a slightly stabilizing impact. One of the most surprising findings for us was that such a subtle change in AR12 led to a gain-of-function phenotype distinctly affecting the polymodal gating of the channel (Fig. 6). Serine is statistically three times less favored than threonine in T/SPLH tetrapeptides^29^, and thus the role of S448 in TRPA1 is likely to be specific and may be evolutionarily fine-tuned. The ^448^SPLH motif, strictly conserved in mammals, birds and reptiles, aligns with SALH in zebrafish, TALH in the nematode worm *Caenorhabditis elegans*, and TPLH in hymenoptera- specific TRPA. A strikingly conserved quadruplet of asparagines followed by glutamate, instead of ^443^SKDKK in human TRPA1, precedes the SPLH motif in insects.

**Figure 5:**
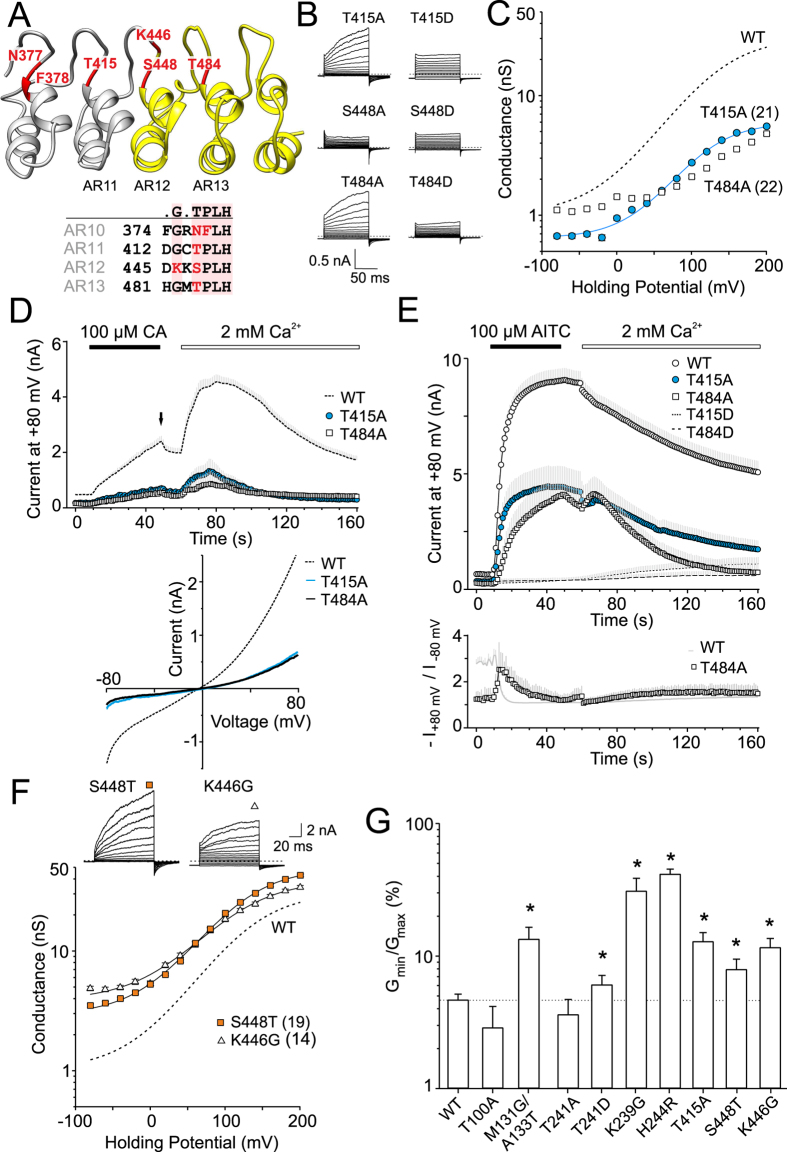
A short range stability of the ankyrin repeat 12 determines the gating and calcium regulation of human TRPA1. (**A**) Homology model of the ankyrin repeats 10 to 14 of hTRPA1. The grey part is based on ankyrinR (pdb code 1N11), the yellow part is based on the published structure of hTRPA1 (13; pdb code 3J9P). (**B**) Representative current traces in response to voltage step protocol. Representative current traces for wild-type TRPA1 are shown in Fig. 2B. (**C**) Average conductances obtained from voltage step protocols as in B. Solid line is the best fit to a Boltzmann function. The dashed line represents the fit obtained for wild-type TRPA1 shown in Fig. 2C. Only the voltage-sensitive mutants are shown; *n* indicated in brackets. (**D**) Time course of average whole-cell currents through the wild-type hTRPA1 and the T415A (*n* = 8) and T484A (*n* = 11) mutants measured at +80 mV (protocol shown in Fig. 2D). The application of 100 μM cinnamaldehyde (CA) and subsequent addition of 2 mM Ca^2+^ are indicated above. Below, average current-voltage relationships of traces measured at times indicated by an arrow in panel above. (**E**) Time course of average whole-cell currents through wild-type and mutant hTRPA1 measured at +80 mV. The application of 100 μM allyl isothiocyanate (AITC) and subsequent addition of 2 mM Ca^2+^ are indicated above. Gray bars indicate S.E.M. from 33 (WT), 16 (T415A) and 10 (T484A) cells. T415D and T484D were insensitive to AITC (*n* = 13 and 10). Below, average rectification of currents through WT and T484A shown above. (**F**) Representative current traces in response to voltage step protocol (as shown in Fig. 2B) recorded in control extracellular solution. Below, average conductances obtained from currents as shown above (*n* indicated in brackets). Solid lines are best fits to a Boltzmann function. The dashed line represents the fit obtained from data for wild-type TRPA1. (**G**) Summary of voltage-independent component of voltage-induced gating for wild-type (*n* = 138) and individual mutants shown in this study (*n* = 7–27). The asterisks indicate significant (*P < 0.05) differences between mutant and wild-type TRPA1.

**Figure 6:**
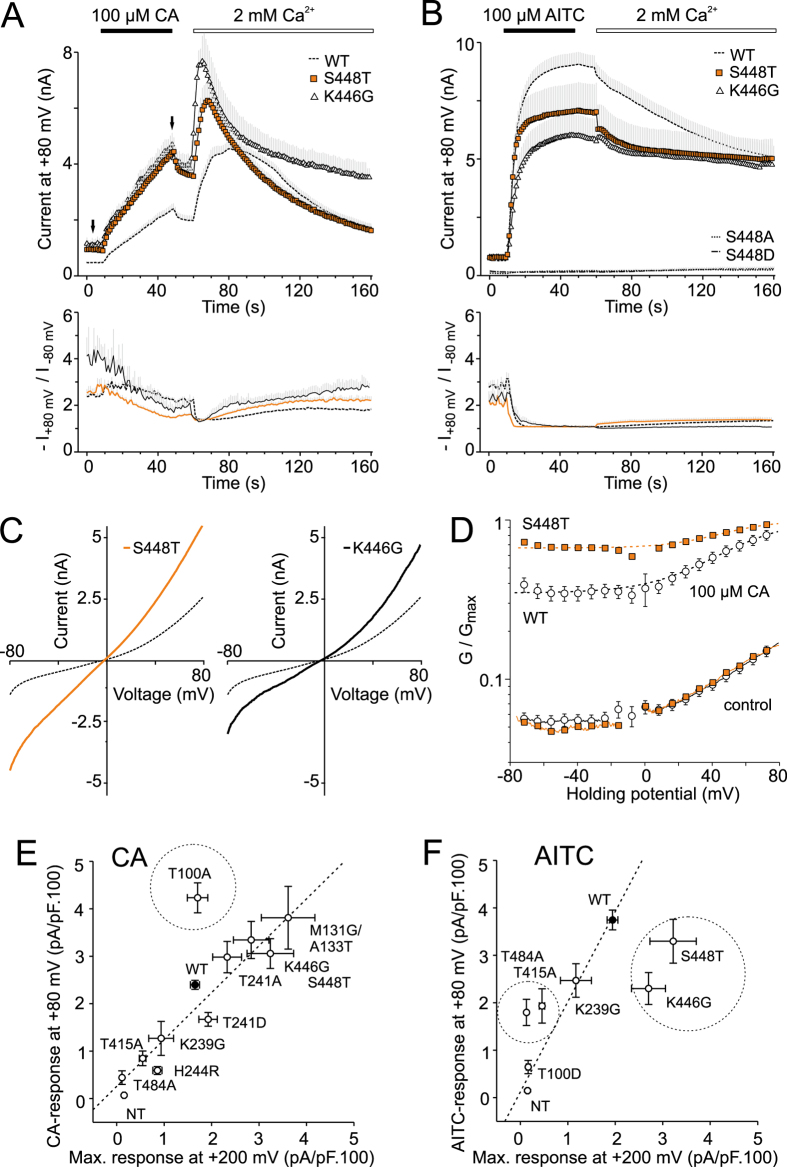
Strict conservations of the T/SPLH motifs in AR11-AR13 are required for functional interactions and not for targeting TRPA1 to the plasma membrane. (**A**,**B**) Time course of average whole-cell currents through wild-type and mutant hTRPA1 measured at +80 mV. The application of 100 μM cinnamaldehyde (CA, in panel (A)) or 100 μM allyl isothiocyanate (AITC, in panel (B)) and subsequent addition of 2 mM Ca^2+^ are indicated above. Below, average rectification of currents shown above. Note, the S448T (*n* = 18) and K446G (*n* = 5) mutants exhibit greater currents than WT in CA but not in the presence of AITC. Mutations S448A and S448D were AITC-insensitive (*n* = 9 and 9). (**C**) Average current-voltage relationships of traces measured after 40 s application of 100 μM CA at time indicated by an arrow in panel A for wild-type (dashed line) and indicated mutants. (**D**) Average conductances obtained for WT (open circles) and for S448T (orange squares) from voltage ramp protocol before (control) and after 40 s application of 100 μM CA at times indicated by arrows in panel A, normalized to *G*_max_. Dashed lines are best fits to a Boltzmann function. The *G*-*V* curves for CA-dependent gating of S448T exhibited significantly different parameters (*V*_50_ = 48 mV, *G*_min_ = 0.67, *z* = 1.5 e_0_, *n* = 14) than wild-type TRPA1 (*V*_50_ = 54 mV, *G*_min_ = 0.35, *z* = 1.2 e_0_, *n* = 91). (**E**) Correlation of the maximum conductances induced by depolarizing voltage (+200 mV; see voltage-step protocol in Fig. 2B) against the maximum outward responses induced by cinnamaldehyde (measured at time indicated by an arrow in Fig. 2D,E) for all responsive mutants and for non-transfected cells (NT; *n* = 14). T100A indicated by a dashed circle diverges from linear relationship. (**F**) Correlation of the maximum current densities induced by depolarizing voltage against the maximum responses induced by AITC. Mutations indicated by dashed circles diverge from linear relationship.

**Figure 7:**
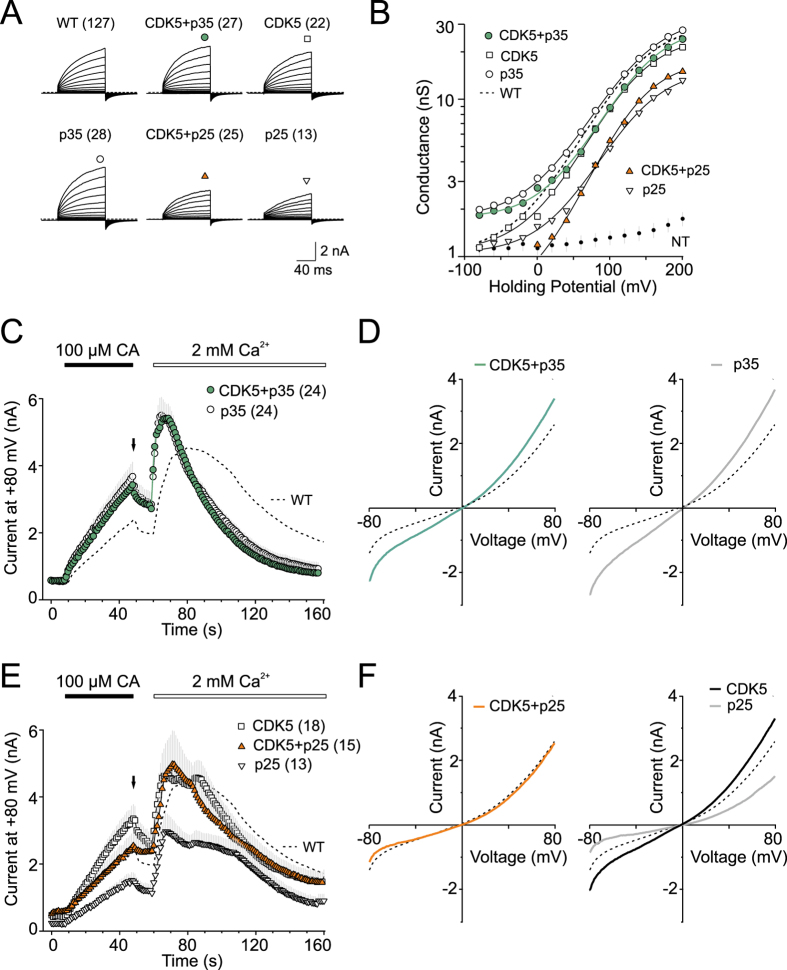
Conserved T/SPLH motifs as putative phosphorylation sites. (**A**) Average current traces in response to indicated voltage step protocol (holding potential −70 mV; 100 ms voltage steps from −80 mV to +200 mV; increment +20 mV) recorded in control extracellular solution ~1 min after whole-cell formation, *n* is indicated in brackets. Note, the presence of p25 protein in the cells had a cytotoxic effect, which resulted in reduced responses of TRPA1. (**B**) Average conductances obtained from voltage step protocols as in A. Solid lines are best fits to a Boltzmann function. The dashed line represents the fit obtained from data for wild-type TRPA1 shown in Fig. 2C. (**C**,**E**) Time course of average whole-cell currents induced by 100 μM cinnamaldehyde (CA) in Ca^2+^-free solution and then exposed to 2 mM Ca^2+^, measured at +80 mV in wild-type (dashed line) and wild-type co-expressed with indicated proteins; *n* is indicated in brackets. The application of CA and subsequent addition of 2 mM Ca^2+^ are indicated above. (**D**,**F**) Average current-voltage relationships of traces measured after 40 s application of 100 μM CA at time indicated by arrows in panels C and E for wild-type (dashed line) and indicated proteins.

**Figure 8:**
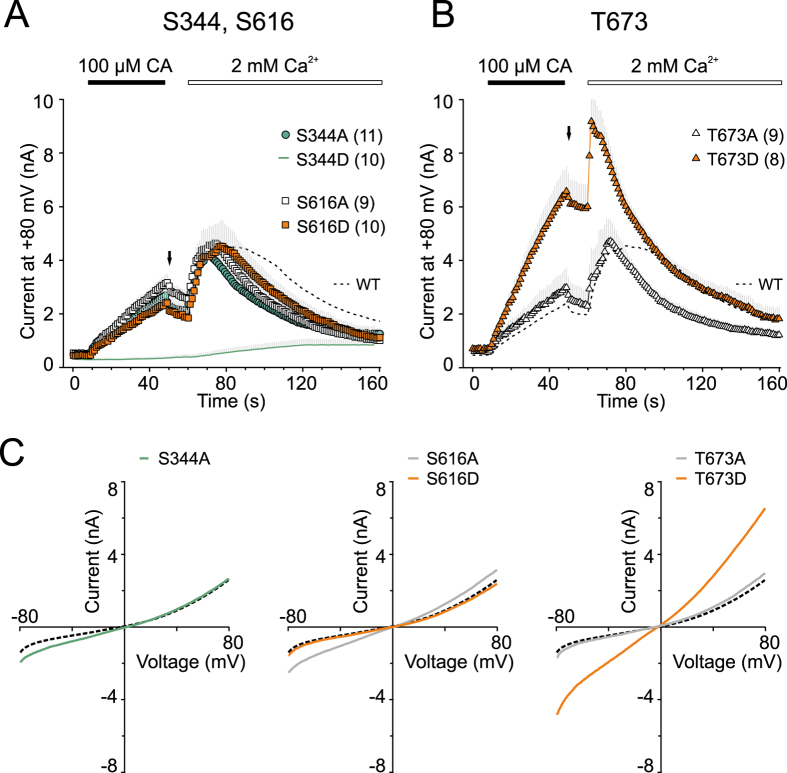
Phospho-mimicking and phospho-null substitutions at S344, S616 and T673. (**A**,**B**) Time course of average whole-cell currents induced by 100 μM cinnamaldehyde (CA) in Ca^2+^-free solution and then exposed to 2 mM Ca^2+^, measured at +80 mV in wild-type (dashed line) and indicated mutants; *n* is indicated in brackets. The application of CA and subsequent addition of 2 mM Ca^2+^ are indicated above. Note, the aspartate mutation of S344 resulted in a loss-of-function phenotype, the T673D mutant is a gain-of-function phenotype. (**C**) Average current-voltage relationships of traces measured after 40 s application of 100 μM CA at times indicated by arrows in panels A and B for wild-type (dashed line) and indicated functional mutants.

We show that strict conservation of the T/SPLH motifs in AR11-AR13 is required for functional interactions, and most likely not for targeting TRPA1 to the plasma membrane. Although changes in plasma membrane expression may also affect the qualitative behavior of the TRPA1 channel^23^,^31^, we believe that the S448T mutation results in a gain-of-function by mainly impacting the transition between the closed and open state of the channel in a way that is independent of the putative voltage sensor (Fig. 5F). When the channel is partially activated by cinnamaldehyde, S448 may contribute to allosteric coupling between the voltage-sensing domain and the gate, as can be inferred from Fig. 6D. To further characterize the impact of the S448T mutation on voltage-dependent gating, we used the allosteric model for voltage-sensor/gate allosteric coupling proposed by^45^,^46^ (Equation 1 in Supplementary Methods). By fitting the normalized *G*-*V* curves for voltage activation, we obtained the values of the equilibrium constant for gate opening L and the allosteric coupling factor D. For wild-type TRPA1, these values were L = 0.06 and D = 166 (Supplementary Fig. S3 and Table S2). The S448T mutation increased the equilibrium constant L by 67% and decreased the allosteric coupling factor D by only 16%. Even more pronounced effects were obtained for K446G, for which L was increased by 166%, whereas D was reduced by 46% compared to the wild-type. Taken together, these findings suggest that an increase in the structural stability of AR12 predominantly impacts the energetics of channel opening and modestly interferes with the allosteric mechanism of coupling the putative voltage-sensing domain and gate opening.

When designing this study, we considered that the strictly conserved T/SPLH motifs are consistent with the consensus phosphorylation sequence of proline-directed Ser/Thr kinases (T/SPXK/H/R). Phosphorylation has been shown to substantially regulate the subcellular targeting, biophysical properties and gating of many TRP channels^47^, to date very little is known about this kind of modification in TRPA1. Therefore for our substitution at the T/S position we used aspartate, which both mimics phosphorylation as well as being anticipated to sufficiently destabilize the ankyrin repeat’s conformation. The data obtained with aspartate mutants compared with their phospho-null counterparts suggest that the strict conservation of the T/SPLH motifs in ARD is not to enable or improve phosphorylation. The only residue fulfilling the consensus requirement and upregulating the function of TRPA1 under phospho-mimicking conditions is T673, which is outside the ARD. Also, the specific result of our study that the co-expression of p35 or CDK5 with p35 significantly increased TRPA1-mediated responses to cinnamaldehyde is an important observation that was not previously reported and is relevant to the putative roles of the N-terminal T/SPXH motifs.

We believe that the information provided by this study will facilitate our future understanding of how TRPA1 operates, how is used for signal transduction and how it is regulated in native cells.

## Methods

### Expression and constructs of hTRPA1 channel

Human embryonic kidney 293T (HEK293T) cells were cultured in Opti-MEM I media (Invitrogen) supplemented with 5% fetal bovine serum as described previously^48^. Cells were transiently co-transfected with 400 ng of cDNA plasmid encoding wild-type (WT) or mutant human TRPA1 (wild type in the pCMV6-XL4 vector, OriGene) and with 200 ng of GFP plasmid (TaKaRa) per 1.6 mm dish using the magnet-assisted transfection (IBA GmbH.) technique. In the experiments with CDK5, the WT was co-expressed with CDK5 and p25 (p35) in the cDNA ratio 250:250:125 (400:250:150). A 1:1 cDNA ratio was used for the co-expression of the WT with CDK5, p25 or p35. The plasmids pcDNA3-CDK5-GFP, pcDNA3.1-P25C-GFP and pCMV-P35 were obtained from the plasmid repository Addgene. The cells were used 24–48 h after transfection. At least three independent transfections were used for each experimental group. The wild-type channel was regularly tested in the same batch as the mutants. The mutants were generated by PCR using a QuikChange Site-Directed Mutagenesis Kit (Stratagene) and confirmed by DNA sequencing (GATC Biotech).

### Electrophysiology

Whole-cell membrane currents were recorded by employing an Axopatch 200B amplifier and pCLAMP 10 software (Molecular Devices). Patch electrodes were pulled from a glass tube with a 1.65-mm outer diameter. The tip of the pipette was heat-polished, and its resistance was 3–5 MΩ. Series resistance was compensated by at least 70% in all recordings. The experiments were performed at room temperature (23–25 °C). Only one recording was performed on any one coverslip of cells to ensure that recordings were made from cells not previously exposed to chemical stimuli. A system for rapid superfusion and heating of the cultured cells was used for drug application^49^. The extracellular bath solutions contained: 150 mM NaCl and 10 mM HEPES, with an added 2 mM HEDTA for the Ca^2+^-free solution, and 2 mM CaCl_2_ for the Ca^2+^-containing solutions, adjusted to pH 7.3 with NaOH, 300 mOsm. The *I-V* relationships were measured in control bath solution containing 160 mM NaCl, 2.5 mM KCl, 1 mM CaCl_2_, 2 mM MgCl_2_, 10 mM HEPES, 10 mM glucose, adjusted to pH 7.3 and 320 mOsm. The whole-cell pipette solution contained the high buffer internal solution: 145 mM CsCl, 5 mM EGTA, 3 mM CaCl_2_, 10 mM HEPES, 2 mM MgATP, pH 7.3, adjusted with CsOH, 290 mOsm. Cinnamaldehyde and allyl isothiocyanate solution was prepared prior to use from a 0.1 M stock solution in Me_2_SO. All of the chemicals were purchased from Sigma-Aldrich.

### Statistical analysis

All of the electrophysiological data were analyzed using pCLAMP 10 (Molecular Devices), and curve fitting and statistical analyses were done in SigmaPlot 10 (Systat Software Inc.). Conductance-voltage (*G-V*) relationships were obtained from steady-state whole cell currents measured at the end of voltage steps from −80 to +200 mV in increments of +20 mV, or from currents recorded by voltage ramp protocol in the presence of 100 μM cinnamaldehyde for 40 s. Voltage-dependent gating parameters were estimated by fitting the conductance *G* = *I*/(*V* − *V*_rev_) as a function of the test potential *V* to the Boltzmann equation: *G* = [(*G*_max_ − *G*_min_)/(1 + exp (−*zF*(*V* − *V*_50_)/*RT*))] + *G*_min_, where *z* is the apparent number of gating charges, *V*_50_ is the half-activation voltage, *G*_min_ and *G*_max_ are the minimum and maximum whole cell conductance, *V*_rev_ is the reversal potential, and *F*, *R*, and *T* have their usual thermodynamic meanings. Statistical significance was determined by Student’s *t-*test or the analysis of variance, as appropriate; differences were considered significant at *P* < 0.05 where not stated otherwise. For statistical analysis of the voltage-independent component of gating (*G*_min_/*G*_max_) data, a logarithmic transformation was used to achieve a normal distribution. The data are presented as means ± S.E.M.

## Additional Information

**How to cite this article**: Hynkova, A. *et al*. N-terminal tetrapeptide T/SPLH motifs contribute to multimodal activation of human TRPA1 channel. *Sci. Rep.* **6**, 28700; doi: 10.1038/srep28700 (2016).

## Supplementary Material

Supplementary Information
