# Supplementary material for: N-terminal tetrapeptide T/SPLH motifs contribute to multimodal activation of human TRPA1 channel
Source: Sci Rep. 2016 Jun 27;6:28700. doi: 10.1038/srep28700 (PMC4922051; doi:10.1038/srep28700)
Supplement: Supplementary Information [file srep28700-s1.pdf]

N-terminal tetrapeptide T/SPLH motifs contribute to multimodal activation of human TRPA1 channel

Supplementary Information

Anna Hynkova, Lenka Marsakova, Jana Vaskova, Viktorie Vlachova*

Department of Cellular Neurophysiology, Institute of Physiology of the Czech Academy of Sciences, Videnska 1083, 142 20 Prague 4, Czech Republic

***Corresponding author:**
Viktorie Vlachova
Department of Cellular Neurophysiology
Institute of Physiology AS CR
Vídenská 1083, 142 20 Prague 4
Czech Republic
Tel. 420-29644-2711
Fax. 420-29644-2488
E-Mail: viktorie.vlachova@fgu.cas.cz

Supplementary Fig. S1

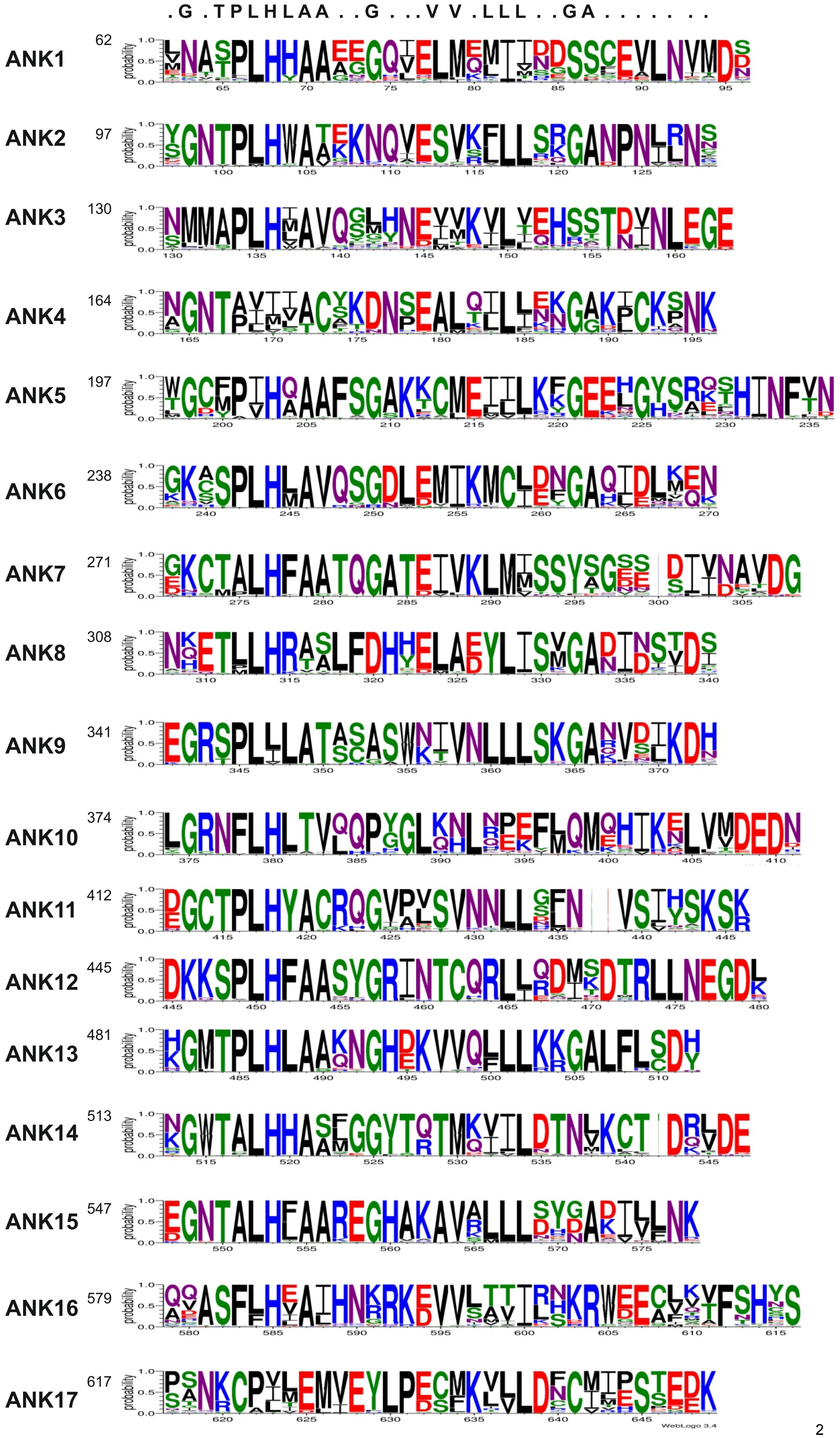

Supplementary Fig. S2

A

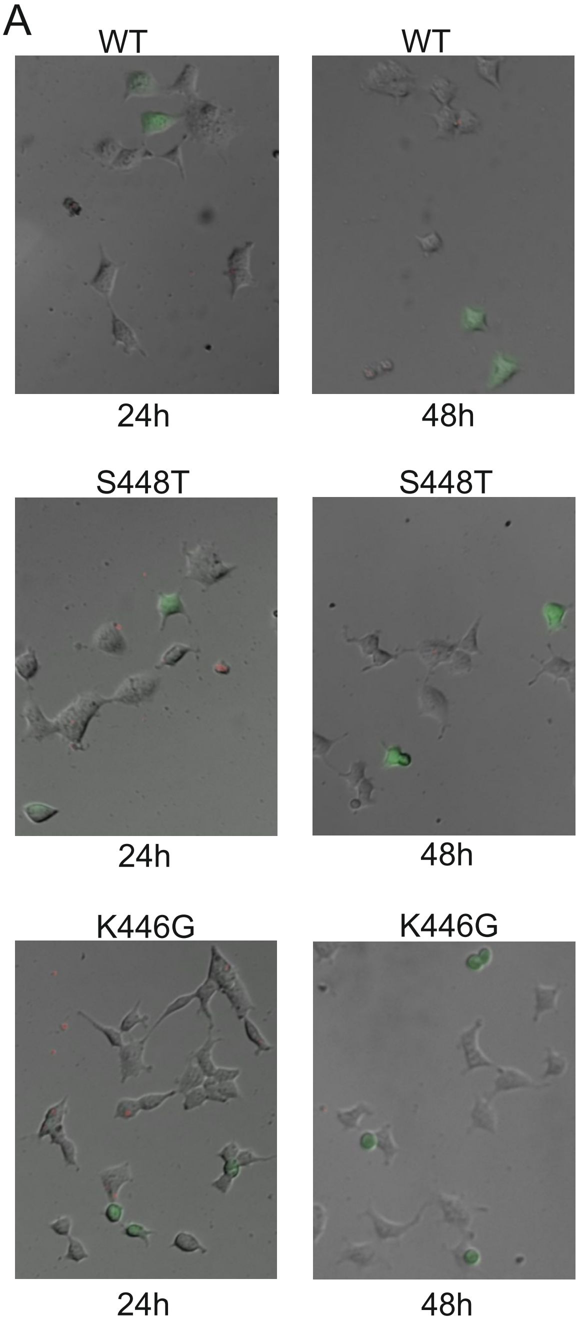

B

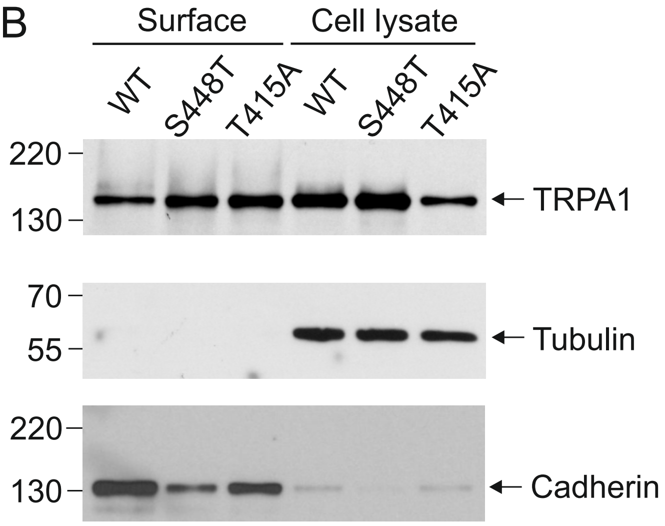

Supplementary Fig. S3

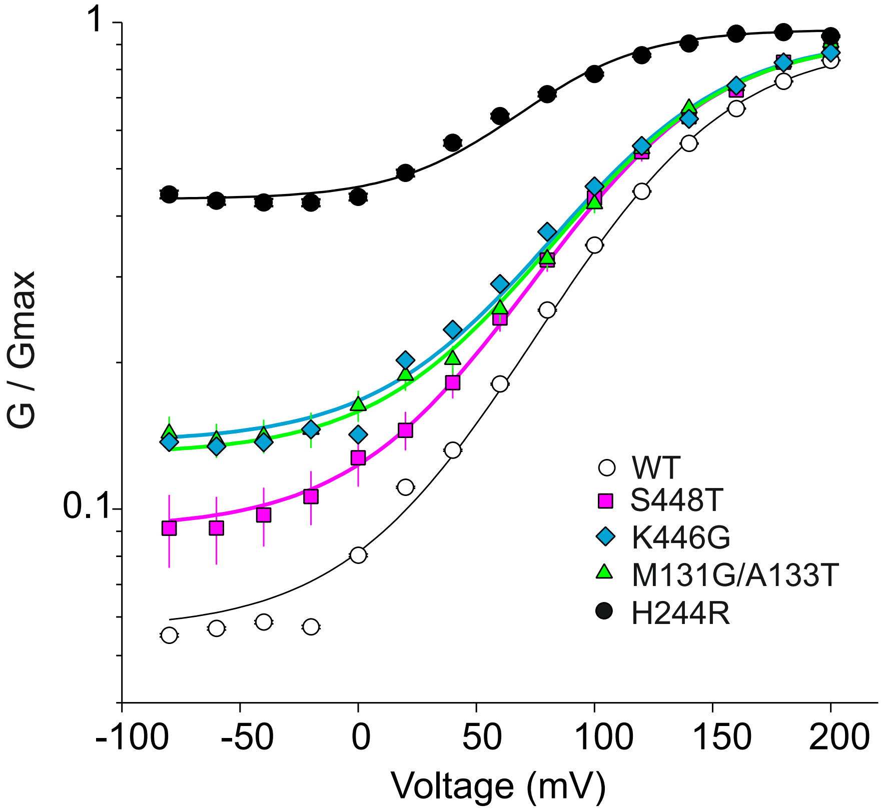

Supplementary Figure S1. Amino acid sequence conservation of the N-termini of TRPA1 proteins represented as a sequence logo generated by a WebLogo server (Crooks et al., 2004). The alignment was obtained from 80 species sequences and is numbered according to human TRPA1. The height of the particular amino acid at each position indicates its probability of occurrence at that position. Above, highly conserved consensus amino acid sequence of the ankyrin repeat proteins. Note the high inter-species conservation of T/SPLH motifs of the ankyrin repeats 2, 6, 11, 12 and 13, and also note a highly conserved and substantial difference from this motif in ankyrin repeat 10 (^{377}NFLH).

Supplementary Figure S2. Cell viability and biotinylation assays. (A) HEK293T cells transfected with TRPA1 constructs together with a GFP-marker plasmid (GFP). Cells were stained with the vital stain propidium iodide (PI) to measure cell viability. Differential interference contrast (DIC) images and overlays of GFP fluorescence and PI staining. Images were collected from randomly chosen fields of cells from three independent experiments after 24 and 48 hours. Representative fluorescence images of propidium iodide (red) and GFP (green) are shown. (B) Representative immunoblots from biotinylation assays using HEK293T cells transfected with WT, S448T and T415A hTRPA1. Band sizes are indicated (in kD) left. Tubulin and pan-cadherin were used as loading controls.

Supplementary Figure S3. The allosteric model predicts behavior of the S448T, K446G, H244R, and M131G/A133T mutants. The normalized *G-V* relationships were fitted to Equation 1. The values of the parameters obtained from fit are presented in Table S2. The modeling indicates that mutations in the conserved T/SPLH motifs in ankyrin repeat domain

impact the energetics of channel opening and interfere with the allosteric mechanism of coupling of putative voltage-sensing domain and gate opening.

Supplementary Methods

Cell viability assay

The cells were incubated with 7 µM propidium iodide (Sigma-Aldrich) in Opti-MEM media supplemented with 5% fetal bovine serum for 30 min at 37 ^{o}C, 5% CO_2. Then cells were washed and analysed in control bath solution (see Methods). Fluorescence images of the cells were recorded with a Cell^R imaging system based on an Olympus IX81 inverted microscope. The excitation light was generated with a Polychrome V polychromator (Till Photonics, Grafelfing, Germany), and the fluorescence emission was detected with a Hamamatsu Orca-ER camera (Hamamatsu Photonics, Hamamatsu City, Japan). The excitation wavelength was 480 ± 7.5 nm (GFP) or 540 ± 7.5 nm (propidium iodide), and emission was collected using 515 nm (GFP) or 575 nm (propidium iodide) long pass filters . Data were collected using Cellˆ R software (Olympus), and the analysis was done using the program ImageJ (National Institutes of Health, Bethesda, MD).

Biotinylation of cell surface proteins

The HEK293T cells were transfected with 1.5 µg of cDNA plasmid encoding wild-type or mutant C-terminally GFP-tagged human TRPA1 (in the pCMV6-AC-GFP vector; OriGene) with Lipofectamine 2000 and cultured in a 6-well plate. At 48 h post-transfection, cells were washed with ice-cold phosphate buffered saline (PBS) buffer three times and incubated with 0.5 mg/ml EZ-link Sulfo-NHS-LC-biotin (Thermo Scientific) in PBS for 30 min at 4 °C. After quenching the reaction (50 mM glycine in PBS), the cells were homogenized and crude plasma membrane fraction was prepared as described by Chaudhury et al. (2011). The membrane fraction was incubated with streptavidin-agarose beads (Thermo Scientific) at room temperature with constant rotation for 2 hours, followed by four washes. The

biotinylated protein fractions and the cell lysate fractions (1.5%) were separated using SDS/PAGE (7% SDS-polyacrylamide), followed by electroblotting onto PVDF membranes (polyvinylidene difluoride, Amersham, Germany). Immunoblots were probed with a mouse anti-GFP (1:2000; OriGene), a mouse anti-tubulin as a cytoplasmic marker (1:1500, Exbio) or mouse anti-pan Cadherin as a plasma membrane marker (1:1000, Abcam) primary antibodies in TBS/Tween for 1 h at room temperature. After 3-times 10 min washes in TBS/Tween immunoblots were incubated (1 h, room temperature) with horseradish peroxidase-conjugated goat anti-mouse IgG secondary antibody (1:20000, Thermo Scientific) followed by 5-times 5 min washes in TBS/Tween. Detection was done with a SuperSignal West Femto Maximum Sensitivity chemiluminiscent substrate (Thermo Scientific). Immunoblots were digitized and quantified with ImageJ 1.41v software (National Institutes of Health).

Molecular modelling and stability change predictions

Structural hypotheses were tested by mapping the residues onto the homology models of the N-terminal ankyrin repeat domain region made using the software Yasara (version 15.9.6)(Krieger and Vriend, 2014) in conjunction with the Swiss-Model and I-Tasser protein modeling servers (Arnold et al., 2006; Kopp and Schwede, 2006; Zhang, 2008; Bordoli et al., 2009; Kiefer et al., 2009; Roy et al., 2010; Yang et al., 2015). For theoretical energy estimations, the program force-field FoldX for calculating the folding energies of proteins and for calculating the effect of a point mutation on the stability of a protein was used as the graphical interface to Yasara (Schymkowitz et al., 2005; Van Durme et al., 2011). Theoretical energy measurements were compared for various models with a satisfactory quality score obtained from both servers. The FoldX plugin calculates the free energy change expressed as $\Delta\Delta G(\text{change}) = \Delta G(\text{MT}) - \Delta G(\text{WT})$, where $\Delta G(\text{WT})$ and $\Delta G(\text{MT})$ are the free energies of the wild type and the mutant (MT). $\Delta\Delta G(\text{change}) > 0$ if the mutation is destabilizing and < 0 if

the mutation is stabilizing. Changes by ± approximately 0.5 kcal/mol are considered insignificant. The ankyrin repeats tested in this study are very consensus-like and the results from calculations on various models were predominantly consistent. Some of the mutations were found to be significantly stabilizing. Calculations for each of the modules AR1-AR3, AR5-AR7, AR10-AR14 shown in Figs. 2, 3 and 5 were performed for the molecular models flanked with at least one N- and one C-terminal capping repeats.

Allosteric gating model predictions for specific mutants

G-*V* curves for voltage-induced gating were normalized to G_{max} estimated from the Boltzmann fit. The allosteric model for channel activation was used, assuming that two independent equilibria interact allosterically (Horrigan and Aldrich, 2002; Brauchi et al., 2004; Brauchi and Orio, 2011). The first equilibrium is between resting and activated state of the voltage sensor, the second between the closed and open conformation of the channel pore gate. *G*-*V* curves were fitted by using the allosteric model :

$$P_O(V) = (1 + ((1 + J)/(L(1 + JD)))^{-1} \qquad \text{Equation 1}$$

where $P_O(V)$ is the probability of being in an open state at a given voltage, J = J_0 exp (zFV/RT), J_0 is the equilibrium constant for voltage-sensor activation at 0 mV, *F, R*, and *T* have their usual thermodynamic meanings, *z* gating valence, L the equilibrium constant for gate opening, D the allosteric coupling factor. Fitting was done in SigmaPlot 10 (Systat Software Inc.).

References:

Arnold, K., L. Bordoli, J. Kopp, and T. Schwede. 2006. The SWISS-MODEL workspace: a web-based environment for protein structure homology modelling. *Bioinformatics*. 22:195-201.

Bordoli, L., F. Kiefer, K. Arnold, P. Benkert, J. Battey, and T. Schwede. 2009. Protein structure homology modeling using SWISS-MODEL workspace. *Nat. Protoc.* 4:1-13.

Brauchi, S., and P. Orio. 2011. Voltage sensing in thermo-TRP channels. *Adv. Exp. Med. Biol.* 704:517-530.

Brauchi, S., P. Orio, and R. Latorre. 2004. Clues to understanding cold sensation: thermodynamics and electrophysiological analysis of the cold receptor TRPM8. *Proc. Natl. Acad. Sci. U S A.* 101:15494-15499.

Chaudhury, S., Bal, M., Belugin, S., Shapiro, M. S. & Jeske, N. A. AKAP150-mediated TRPV1 sensitization is disrupted by calcium/calmodulin. *Mol. Pain* **7**, 34 (2011).

Crooks, G.E., G. Hon, J.M. Chandonia, and S.E. Brenner. 2004. WebLogo: A sequence logo generator. *Genome Res.* 14:1188-1190.

Horrigan, F.T., and R.W. Aldrich. 2002. Coupling between voltage sensor activation, Ca2+ binding and channel opening in large conductance (BK) potassium channels. *J. Gen. Phys*. 120:267-305.

Kiefer, F., K. Arnold, M. Kunzli, L. Bordoli, and T. Schwede. 2009. The SWISS-MODEL Repository and associated resources. *Nucleic Acids Res.* 37:D387-D392.

Kopp, J., and T. Schwede. 2006. The SWISS-MODEL repository: new features and functionalities. *Nucleic Acids Res.* 34:D315-D318.

Krieger, E., and G. Vriend. 2014. YASARA View - molecular graphics for all devices - from smartphones to workstations. *Bioinformatics*. 30:2981-2982.

Roy, A., A. Kucukural, and Y. Zhang. 2010. I-TASSER: a unified platform for automated protein structure and function prediction. *Nat. Protoc.* 5:725-738.

Schymkowitz, J., J. Borg, F. Stricher, R. Nys, F. Rousseau, and L. Serrano. 2005. The FoldX web server: an online force field. *Nucleic Acids Res.* 33:W382-388.

Van Durme, J., J. Delgado, F. Stricher, L. Serrano, J. Schymkowitz, and F. Rousseau. 2011. A graphical interface for the FoldX forcefield. *Bioinformatics*. 27:1711-1712.

Yang, J., R. Yan, A. Roy, D. Xu, J. Poisson, and Y. Zhang. 2015. The I-TASSER Suite: protein structure and function prediction. *Nat. Methods*. 12:7-8.

Zhang, Y. 2008. I-TASSER server for protein 3D structure prediction. *BMC Bioinformatics*. 9:40.

Supplementary Table S1

Phosphorylation site predictions for hTRPA1 and CDK5

	**PREDIKIN** for CDK5	**Kinase Phos2** HMM bitsc/E-value	**NetPhosK 2**	**ScanSite 3** Score/ Percentile	**PPSP** Risk-Diff.	**GPS 3.0** Medium	**Phosida** Stringency pS/pT	**MUSITE**	**MIMP/** CDK5	**NetPhorest CDK1/ 3/5**	**DISPHOS**
**T100**	72.20	-7.5/ 130	0.361	0.571/ 2.683%	8.05	14.6	65%	1.31 95.57%	0.874	0.24	0.360
**T241**	71.79	-2.8/ 34	0.315	0.578/ 2.869%	7.64	13.7	70%	1.20 94.83%	0.882	0.23	0.278
**S344**	74.63	-	0.497	0.672/ 6.219%	6.94	11.6	95%	1.19 94.73%	0.902	0.12	0.503
**T415**	72.94	-	0.045	0.571/ 2.683%	6.52	22.3	70%	1.54 97.83%	0.874	0.24	0.759
**S448**	70.52	-	0.959	0.433/ 0.497%	6.60	18.2	95%	1.23 95.01%	0.903	0.34	0.616
**T484**	68.92	-1/ 17	0.119	0.543/ 2.004%	6.23	20.1	65%	1.36 96.03%	0.884	0.27	0.456
**S616**	79.77	-2.3/ 27	0.848	0.546/ 2.082%	7.75	9.3	95%	0.95 93.82%	0.866	0.27	0.220
**T673**	74.75	-4.7/ 66	0.535	0.616/ 4.113%	7.46	11.0	90%	1.31 95.57%	0.877	0.19	0.527
**T448**	68.61	-6.4/ 100	0.144	0.433/ 0.497%	5.97	19.9	85%	1.19 94.73%	0.903	0.34	0.462
**hTRPV1 T407**	86.00	1.9/ 5.4	0.215	0.432/ 0.483%	8.78	19.2	90%	1.49 97.33%	0.93	0.34	0.686

Supplementary Table 1. Predictions for putative phosphorylation sites in human TRPA1. For comparison, the score for each prediction is shown for human TRPV1 in the last line.

References: Predikin: http://predikin.biosci.uq.edu.au, KinasePhos: H.D. Huang*, T.Y. Lee, S.W. Tseng, and J.T. Horng. (2005) "KinasePhos: a web tool for identifying protein kinase-specific phosphorylation sites" Nucleic Acids Research, Vol. 33, W226-229.
NetPhosK2.0: http://www.cbs.dtu.dk/services/NetPhos/, ScanSite3: http://scansite3.mit.edu/, PPSP: http://ppsp.biocuckoo.org/, GPS: http://gps.biocuckoo.org/, PHOSIDA 2011: the posttranslational modification database', Florian Gnad, Jeremy Gunawardena, Matthias Mann (2011); Nucleic Acids Research.
MUSITE: Jianjiong Gao, Jay J. Thelen, A. Keith Dunker, and Dong Xu. Musite, a Tool for Global Prediction of General and Kinase-Specific Phosphorylation Sites. Molecular & Cellular Proteomics. 2010. 9(12):2586-600. MIMP: Wagih, O., Reimand, J., and Bader, G. D. (2015)

MIMP: predicting the impact of mutations on kinase-substrate phosphorylation, Nat. Methods 12, 531-533.
NetPhorest: Horn et al., KinomeXplorer: an integrated platform for kinome biology studies. Nature Methods 2014 Jun;11(6):603–4.
Miller et al., Linear Motif Atlas for Phosphorylation-Dependent Signaling. Science Signaling, 2 September 2008, Vol 1, Issue 35, p. ra2.
DISPHOS 1.3: http://www.dabi.temple.edu/disphos/

Supplementary Table S2

	J_0	z	L	D	ΔG_0 kcal/mol
WT	0.003	0.73	0.0599	166.00	2.0503
S448T	0.003	0.64	0.1001	139.30	1.8539
K446G	0.003	0.66	0.1590	89.94	1.8392
M131G-A133T	0.003	0.70	0.1487	93.15	1.8577
H244R	0.003	0.95	0.7659	38.73	1.4161

Supplementary Table 2. Allosteric model parameters for S448T, K446G, and M131G/A133T, and H244R. *G-V* curves were fitted by using the allosteric model described by Equation 1, where J_0 is the equilibrium constant for voltage-sensor activation at 0 mV, z gating valence, L the equilibrium constant for gate opening, D the allosteric coupling factor.
